# A bibliometric analysis of acupuncture applied to primary osteoporosis

**DOI:** 10.3389/fmed.2025.1623239

**Published:** 2026-01-06

**Authors:** Guangbin Yu, Hongyuan Song, Yingying Gao, Guizhen Chen, Yunxiang Xu

**Affiliations:** 1 Shenzhen Bao’an Traditional Chinese Medicine Hospital, Guangzhou University of Chinese Medicine, Shenzhen, Guangdong, China; 2 Clinical Medical School of Acupuncture, Moxibustion and Rehabilitation, Guangzhou University of Chinese Medicine, Guangzhou, Guangdong, China

**Keywords:** acupuncture, primary osteoporosis, CiteSpace, VOSviewer, postmenopausal osteoporosis

## Abstract

**Purpose:**

Recent research suggests that acupuncture holds significant potential in the treatment of primary osteoporosis. However, a comprehensive bibliometric analysis on this topic has not yet been conducted. Therefore, the aim of this study is to investigate the research hotspots related to acupuncture for primary osteoporosis.

**Methods:**

The Web of Science Core Collection database was searched for relevant publications from 2000 to 2025. Countries, institutions, authors, keywords, and literature were analyzed and visualized using bibliometric software, including CiteSpace 6.4.R1, VOSviewer 1.6.20, BICOMB, gCLUTO, SPSS 27.0, and Microsoft Charticulator, to investigate scientific achievements, research collaboration networks, research hotspots, and research trends.

**Result:**

This study analyzed 775 publications. “Osteoporosis International” is the most-cited journal, and the United States of America conducts the most research output and impact. Guangzhou University of Chinese Medicine is the most prolific institution studying acupuncture for primary osteoporosis; Cortet Bernard and Kanis JA are the authors with the most relevant literature and citations in this field. Keyword analysis revealed that the most frequently occurring term. Additionally, the analysis of the keyword analysis showed that the keyword with the highest number of occurrences was postmenopausal women; The results of the keyword and literature analysis indicate that postmenopausal women, osteoporosis-related complications, Traditional Chinese Medicine, and osteoporosis prevention are emerging as new research hotspots. This bibliometric study provides valuable insights into the research status and trends in acupuncture for primary osteoporosis over the past two decades.

**Conclusion:**

Supported by high-quality research, acupuncture is increasingly recognized as an effective treatment for primary osteoporosis. Future research should focus on postmenopausal women, osteoporosis-related complications, Traditional Chinese Medicine, and the prevention of osteoporosis.

## 1 Introduction

Osteoporosis is a metabolic bone disorder characterized by low bone mass and altered bone microarchitecture, which increases the risk of fractures (1). According to the World Health Organization (WHO) criteria, osteoporosis is defined as a bone mineral density (BMD) that is 2.5 standard deviations or more below the average value for young, healthy women, corresponding to a T-score of less than -2.5 SD (2). Clinically, osteoporosis is associated with pain—particularly in the spinal region—and, in severe cases, may lead to spinal deformities and fragility fractures. Additionally, blood levels of calcium, phosphorus, alkaline phosphatase, and parathyroid hormone often exhibit abnormal fluctuations. Osteoporosis is categorized into primary and secondary types, depending on its underlying causes. Primary osteoporosis includes postmenopausal osteoporosis, senile osteoporosis, and idiopathic osteoporosis. Postmenopausal osteoporosis typically occurs in women within 5–10 years following menopause; senile osteoporosis refers to osteoporosis that develops after the age of 70; idiopathic osteoporosis primarily affects adolescents, and its etiology remains unknown (3, 4). The prevalence of primary osteoporosis varies by country; for instance, researchers estimate that 10.2 million adults aged over 50 in the United States have osteoporosis (5), while in Canada, more than 2 million individuals are living with the condition (6).

Furthermore, recent studies indicate that, in addition to the high prevalence among older adults, the incidence of osteoporosis is significantly increasing among children and adolescents (7). Beyond its high prevalence, osteoporosis also incurs substantial economic costs for countries worldwide. For example, recent data from China indicate that the median per-admission inpatient cost for osteoporotic fractures is ¥18,587 (8). In Mexico, the direct cost associated with more than 75,000 fragility fractures was estimated at $256.2 million in 2010, with a projected growth of 41.7% expected by 2020 (9).

Primary osteoporosis is currently treated using various therapeutic methods, with drug therapy primarily focused on calcium (10), vitamin D (11), and estrogen (12, 13). Additionally, Traditional Chinese Medicine (TCM), including moxibustion (14, 15) and Chinese herbal medicine (16–18), has gradually attracted the attention of researchers in the context of primary osteoporosis (19–22). Numerous studies have demonstrated that acupuncture can significantly increase bone mineral density (BMD) at multiple sites, such as the lumbar spine, femoral neck, and tibia; elevate levels of estradiol (E2) and superoxide dismutase (SOD) activity; regulate bone metabolism-related markers including osteocalcin (OC), procollagen type I N-terminal propeptide (PINP), bone-specific alkaline phosphatase (B-ALP), and β-cross-linked C-telopeptide of type I collagen (β-CTX); and also alleviate pain, improve dysfunction, and enhance quality of life (23–26). However, the relatively fragmented nature of research on acupuncture for primary osteoporosis makes it challenging for clinicians and researchers to effectively understand the current state of the field and identify emerging trends. Therefore, this study will use bibliometric tools to collect literature data from the Web of Science (WoS) Core Collection database, enabling clinicians and researchers in acupuncture for primary osteoporosis to stay informed about research trends and better determine the discipline’s future direction.

## 2 Methods

### 2.1 Materials and methods

#### 2.1.1 Data sources and search strategies

In this study, the WoS core collection database, which has the longest coverage time and high-quality database, was selected as the source of available databases (27–29). Relevant literature was retrieved from the Web of Science Core Collection using the following Search strategy: TS = [(senile osteoporosis OR postmenopausal osteoporosis OR primary osteoporosis OR POP) AND (acupuncture OR electroacupuncture OR warm needling OR fire needling OR pressing needling OR transcutaneous electrical acupoint stimulation OR acupoint catgut embedding OR acupoint injection OR Electric acupuncture OR Electroacupuncture therapy OR Electric acupuncture therapy OR EA)]. Therapies related to acupoints defined in a non-traditional way, such as auricular acupuncture and wrist-ankle acupuncture, will be excluded. This search strategy was limited to published English papers between January 2000 and August 2025. The final results were saved as a plain text file with full records and cited references.

#### 2.1.2 Inclusion and exclusion criteria

Inclusion criteria were: (1) articles published from 2000 to 2025; (2) articles retrieved from the Web of Science; (3) only articles and reviews were included.

**FIGURE 1:**
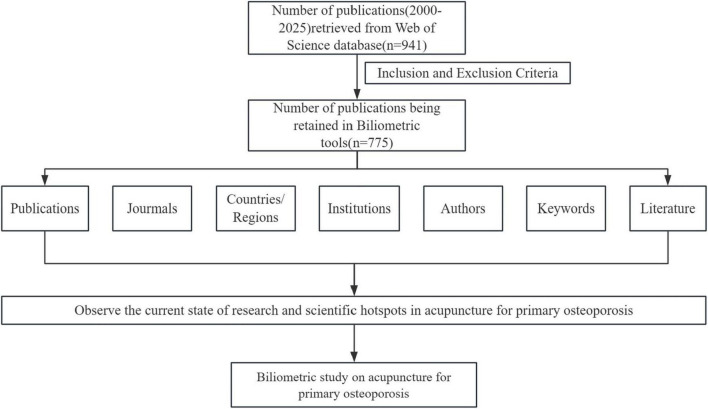
Flowchart depicting the article selection process.

Exclusion criteria were: (1) articles not officially published; (2) unrelated articles; (3) non-English documents. The search process is illustrated in Figure 1.

#### 2.1.3 Bibliometric methods and analysis tools

In this study, publications of acupuncture for primary osteoporosis published from 2000 to 2025 were retrieved from the Web of Science, to explore, using the topic search. Multiple bibliometric tools were employed to ensure a comprehensive analysis. The main visualization software included CiteSpace 6.4.R1 and VOSviewer 1.6.20. CiteSpace, which is based on the Java platform, was used to visualize and analyze journals, countries, institutions, authors, keywords, and references. The parameters in CiteSpace were configured as follows: Time Slicing was set from 2000 to 2025, with Years Per Slice equal to 5; all Term Sources were selected by default; Node Types were chosen sequentially, and the “Top N” value was set to 50. VOSviewer was utilized for the visualization and analysis of journals and keywords. For keyword analysis, the minimum occurrence threshold was set to 3, and for journal co-citation analysis, the minimum co-citation count was set to 5. Additionally, BICOMB (Bibliographic Items Co-occurrence Matrix Builder, version 2.01, developed by China Medical University) served as a core bibliometric tool. It performed three major functions: preprocessing the retrieved 775 articles from Web of Science Core Collection by extracting keywords, authors, and institutions to build a standardized co-occurrence matrix that was used as input for Gcluto (Graphical Clustering Toolkit) (30); working with SPSS 27.0 to generate initial centrality and density values required for strategic coordinate mapping; and assisting in the quadrant division of research themes. Specific parameters were applied: keywords were filtered by frequency ≥ 3, authors and institutions were included if they had published at least 2 relevant articles. The Ochiai coefficient was used to calculate co-occurrence strength (threshold ≥ 0.1). The strategic coordinate map was generated using k-means clustering with 5 clusters. gCLUTO was applied to perform clustering and visualization based on the matrix data from BICOMB. The clustering method used was agglomerative hierarchical clustering. An intra-cluster similarity threshold ≥ 0.6 was adopted to ensure thematic consistency within clusters. The aim was to achieve high intra-cluster similarity and significant inter-cluster differentiation, leading to the identification of thematic clusters such as cellular-level research and spinal rehabilitation, which helped clarify research subfields. Finally, Microsoft charticulator is mainly used to generate international collaboration maps among countries.

## 3 Results

### 3.1 Analysis of publications

**FIGURE 2:**
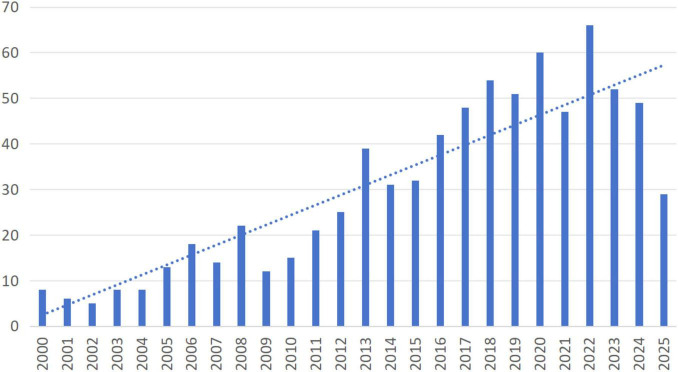
Annual publications of acupuncture for primary osteoporosis.

A total of 775 publications were included in this study, with an average of approximately 29.8 publications per year. As illustrated in Figure 2, the treatment of primary osteoporosis through acupuncture can be categorized into three distinct periods based on the fluctuations in the number of publications over the years. The first period, referred to as the slow development phase (2000–2004), had an average annual output of 7 papers, reflecting a low volume of publications. The second period, known as the stable development phase (2005–2015), saw an average annual output of 20.1 papers, indicating a significant increase in the number of publications compared to the first phase. During this period, the annual output of papers exhibited less variability. The third phase, termed the rapid development period (2016–2025), recorded an average annual output of 45.33 papers, demonstrating a substantial increase in the number of publications (31–37). Although there were slight decreases in the number of papers in certain years, the overall trend indicates that, over time, research and findings related to acupuncture for primary osteoporosis have been on the rise, leading to the emergence of new research hotspots.

### 3.2 Analysis of journals

**FIGURE 3:**
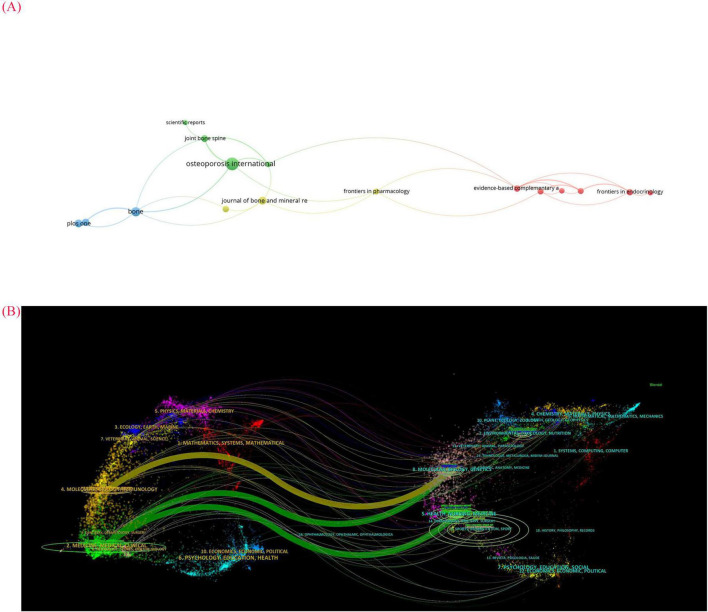
**(A)** The network visualization map of journal co-citation analysis by VOSviewer. The larger circle in the graph means that the journal corresponding to that circle has been cited more often and has more influence in the field of acupuncture for primary osteoporosis. **(B)** Dual-map overlay of journals related to Acupuncture for primary osteoporosis. The left side of the dual map is citing journals while the right side is cited journals, and the line in the middle indicates the association between them.

In this study, we utilized VOSviwer to generate a journal co-citation analysis (Figure 3A) and CiteSpace 6.4.R1 to create a dual-map overlay of journals (Figure 3B). We summarized the five most influential journals in the field of acupuncture for the treatment of primary osteoporosis. As shown in Table 1, the top four journals are high-impact publications in orthopedics. Among these five journals, *Osteoporosis International* is the most influential, followed by *Journal of Clinical endocrinology and metabolism* (ranked second) and *Journal of Bone and Mineral Research* (ranked third), both of which are associated with a significant number of publications. The journal dual-map overlay analysis was proposed by Professors Chaomei Chen and Jie Li and can be used to study the distribution of disciplines corresponding to the journals. The results of the journal dual-map overlay are shown in Figure 3B. The distribution of citing journals on the left side represents the disciplines (knowledge carriers) of acupuncture research output for primary osteoporosis, primarily in medicine, medical, and clinical. Conversely, the distribution of cited journals on the right side represents the knowledge sources of acupuncture research findings, mainly in health, nursing, and medicine (38–41). The color pathways in the middle illustrate the relationship between the citing and cited journals.

**TABLE 1:** Top five cited journals related to acupuncture for primary osteoporosis.

Rank	Citations	Journal	IF (2025)	Category quartile
1	854	Osteoporosis International	5.4	Q1
2	628	Journal of Clinical Endocrinology and Metabolism	5.1	Q1
3	620	Journal of Bone and Mineral Research	5.9	Q1
4	599	Bone	3.6	Q2
5	434	Joint Bone Spine	4.3	Q1

### 3.3 Analysis of countries/regions

**FIGURE 4:**
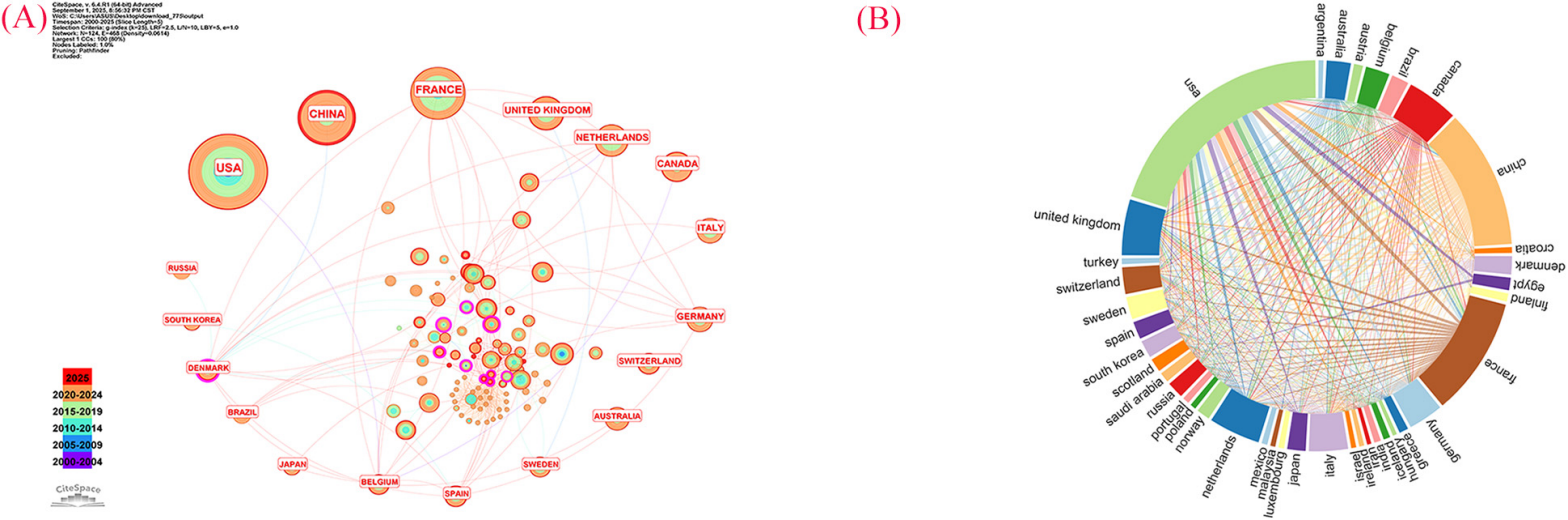
**(A)** Visualization map of countries involved in Acupuncture Therapy for primary osteoporosis (CiteSpace). **(B)** Cooperation network of prolific countries/regions.

In this study, CiteSpace 6.4.R1 was utilized to analyze the volume of literature and identify the most influential countries in the field of acupuncture for primary osteoporosis. As illustrated in Figure 4A, the USA, China, France, the United Kingdom, and the Netherlands emerged as the five countries with the highest research output. The five countries with the most research output and their respective number of publications are detailed in Table 2. As shown in Figure 4B, international collaboration in the field of acupuncture treatment for primary osteoporosis remains fragmented at present.

**TABLE 2:** Top five publications of countries and institutions of acupuncture for primary osteoporosis.

Rank	Count	Country	Rank	Count	Institution
1	290	USA	1	76	Guangzhou University of Chinese Medicine
2	167	China	2	64	Institut National de la Sante et de la Recherche Medicale
3	143	France	3	58	University of California System
4	67	UK	4	49	Harvard University
5	62	Netherlands	5	44	Universite de Lille

Considering the number of publications, international collaborations, and the impact of traditional acupuncture therapy, the USA stands out as the country with the most significant influence in this field.

### 3.4 Analysis of institutions

**FIGURE 5:**
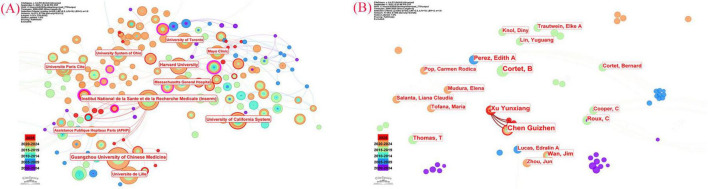
Network map showing the relations between various institutions **(A)** and the authors **(B)**.

The network density of CiteSpace 6.4.R1 can reveal the current level of collaboration among institutions. A higher network density in CiteSpace 6.4.R1 indicates stronger cooperation among these institutions. CiteSpace 6.4.R1 was utilized to generate a plot featuring 373 nodes and a density of 0.0113, as illustrated in Figure 5A. The nodes and densities depicted in the plot indicate a lack of collaboration among research institutions in the field of acupuncture for primary osteoporosis. As shown in Table 2, Guangzhou University of Chinese Medicine is the institution with the highest number of publications and the greatest influence in this area.

### 3.5 Analysis of authors

The co-authorship analysis graph presented in Figure 5B was generated using CiteSpace 6.4.R1. The connecting lines illustrate the degree of collaboration among different authors. As depicted in Figure 5B, there is limited collaboration among researchers in the field of acupuncture for primary osteoporosis, and there is a notable absence of multinational collaboration. Currently, there are 364 authors who have published in this field, with the top five authors listed in Table 3. The most prolific author is Cortet Bernard from the University of Lille, whose primary research focus in recent years has centered on managing the quality of life for postmenopausal women with osteoporosis (42–45). The most cited author is Kanis JA, with 76 citations, recent research has primarily focused on osteoporotic fractures and the treatment and prevention of osteoporosis in elderly patients (46–51). Although the research of Cortet Bernard and Kanis JA does not directly correlate with acupuncture treatment for primary osteoporosis, their research findings have been extensively cited by researchers in this field, making them among the most influential authors in the field.

**TABLE 3:** Top five productive authors and co-cited authors of acupuncture for primary osteoporosis.

Author	Publications	H-index	Co-cited author	Cited times	H-index
Cortet B	10	42	Kanis JA	76	36
Chen Guizhen	10	7	Cummings SR	58	80
Thomas T	8	34	Black DM	50	105
Xu Yunxiang	8	10	Cosman F	37	28
Perez Edith A	7	90	Eastell R	33	10

### 3.6 Analysis of keywords

**FIGURE 6:**
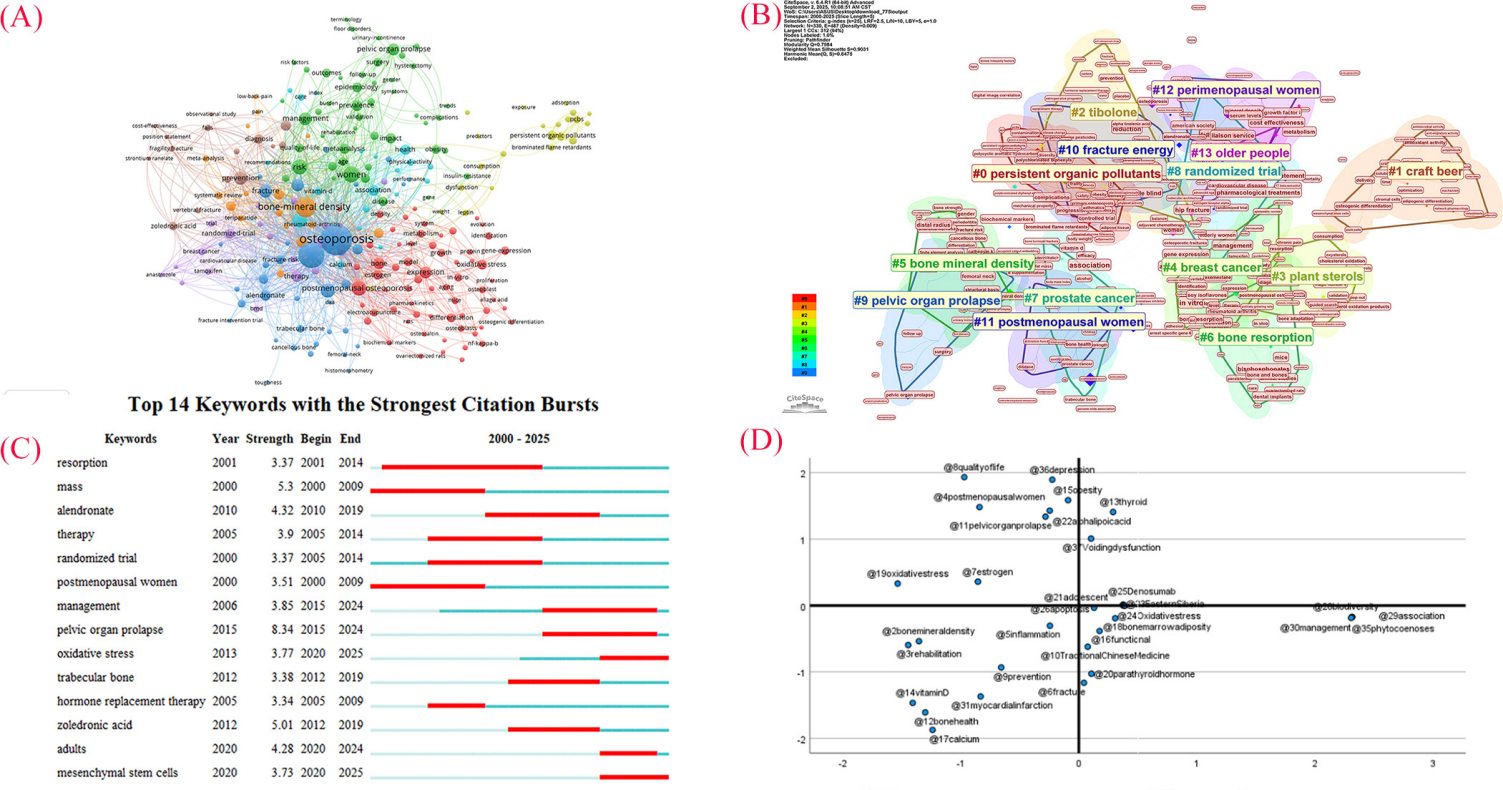
**(A)** Visualization graph of keywords of acupuncture for primary osteoporosis (VOSviewer). **(B)** Visualization graph of keywords of acupuncture for primary osteoporosis (CiteSpace). **(C)** Reference with the strongest citation bursts of Acupuncture in primary osteoporosis. **(D)** Strategic diagram of clusters.

The central idea of the article, as well as the research area, is reflected in the keywords. The use of keyword analysis is beneficial for studying hotspots in the field of acupuncture for primary osteoporosis. We combined the results from Figures 6A,B and summarized them in Table 4. Based on the top five keywords with the highest frequency and centrality, we can infer that research hotspots for acupuncture treatment of primary osteoporosis will focus on: postmenopausal women and the sequelae of osteoporosis.

**TABLE 4:** Keywords in the acupuncture treaty on primary osteoporosis.

Rank	Keyword	Count	Rank	Keyword	Centrality
1	Postmenopausal women	150	1	*In vitro*	0.23
2	Bone mineral density	125	2	Hip fracture	0.20
3	Osteoporosis	90	3	Women	0.19
4	Women	53	4	Double blind	0.17
5	Risk	51	5	Age	0.17

We explored keywords with significant citation bursts using CiteSpace 6.4.R1 and identified the top fourteen (Figure 6C). As shown in Figure 6C, pelvic organ prolapse is the keyword with the strongest outbreak potential. This condition is often associated with osteoporotic fractures (52, 53), indicating that osteoporosis-related sequelae represent a research area with significant potential.

As shown in Figure 6D, the strategic coordinate graph is a two-dimensional coordinate axis, which is the main method of cluster analysis, the X-axis is centripetal degree, which indicates the strength of the interactions between the fields, the greater the strength of the links between different fields, it means that the cluster theme tends to be central in the whole discipline research; the Y-axis is the density, which indicates the strength of the linkages within the cluster theme, the higher the density means that the cluster theme is more mature in its development. In this figure, the first quadrant is the core research theme area, which is the area that researchers in this field focus on, such as voiding dysfunction, thyroid, etc.; the second quadrant is the independent research theme area, which indicates that the keywords are closely connected and mature, but weakly connected with other groups and in a non-core position, belonging to a relatively independent research theme, such as, postmenopausal woman, quality of life, pelvic organ prolapse, etc. The third quadrant is the marginal research theme area, which has a low density and centripetal degree of keywords, indicating that it is internally loose and immature, and has fewer associations with other themes, such as, bone mineral density, rehabilitation, prevention, etc. The fourth quadrant is the potential research theme area, in which the keyword clusters have low density and loose internal structure, but have a high centripetal degree, such as, functional, Traditional Chinese Medicine, parathyroid hormone, etc.

**FIGURE 7:**
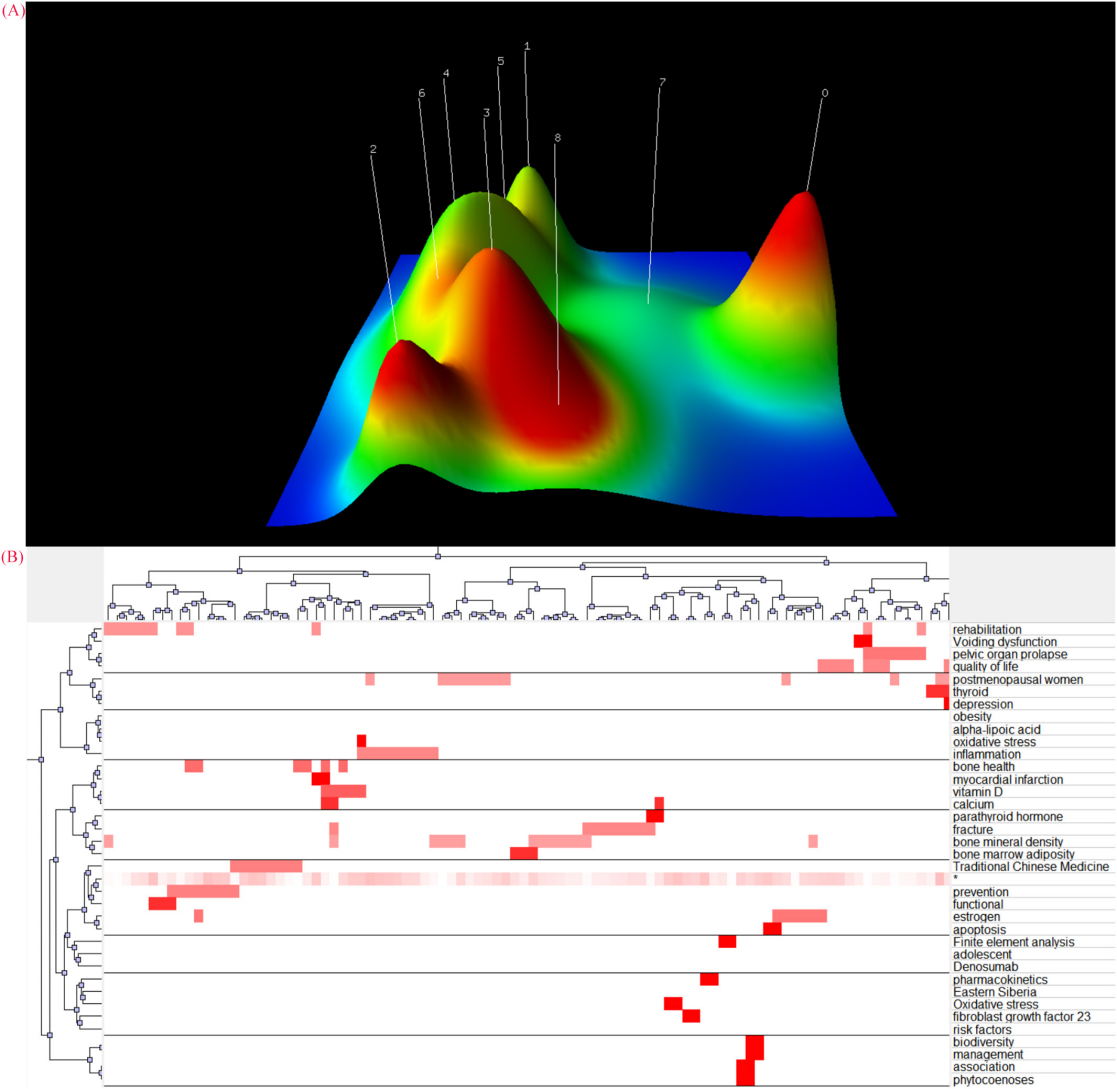
**(A)** Mountain visualization of biclustering analysis results of high-frequency keywords about acupuncture for primary osteoporosis. The clustering results were mainly shown in the visual hill map with different heights and colors, and the height of the hill was proportional to the average similarity of the clusters, the greater the similarity, the steeper the hill. The color of the top of the hill represents the standard deviation of the cluster, where red is low and blue is high, the redder the top of the hill is, the more concentrated the study content is within the cluster. **(B)** Matrix visualization of biclustering analysis results of high-frequency keywords about acupuncture for primary osteoporosis.

Using the gCLUTO software, we mapped the relationship between source articles and keywords through the visual hill map (Figure 7A) and the visual matrix (Figure 7B). In the hill map, the height of the hill corresponds to the intraclass similarity, while the volume reflects the number of objects within the class. The color of the hills indicates the intraclass standard deviation, with red representing a low standard deviation and blue indicating a high standard deviation. Only the color of the peaks is significant. From Figure 7A, we can notice that the tops of clusters 0, 2, and 3 are shown in red, while the tops of the remaining clusters are yellow. This indicates that the overall standard deviation of intra-cluster similarity is low, suggesting that the clustering results obtained are relatively satisfactory.

Cluster 0 conveys a focus on postmenopausal women, particularly concerning related disorders of the endocrine system and psychological conditions.

Cluster 2 expressed attention to osteoporosis-related complications such as fractures.

Cluster 3 reflects an emphasis on Traditional Chinese Medicine and the prevention of osteoporosis.

By integrating the findings from keyword analysis, strategic coordinate mapping, and visual hill mapping, we can conclude that the research hotspots in the field of acupuncture for primary osteoporosis will concentrate on postmenopausal women, osteoporosis-related complications, Traditional Chinese Medicine, and the prevention of osteoporosis.

### 3.7 Analysis of literature

**FIGURE 8:**
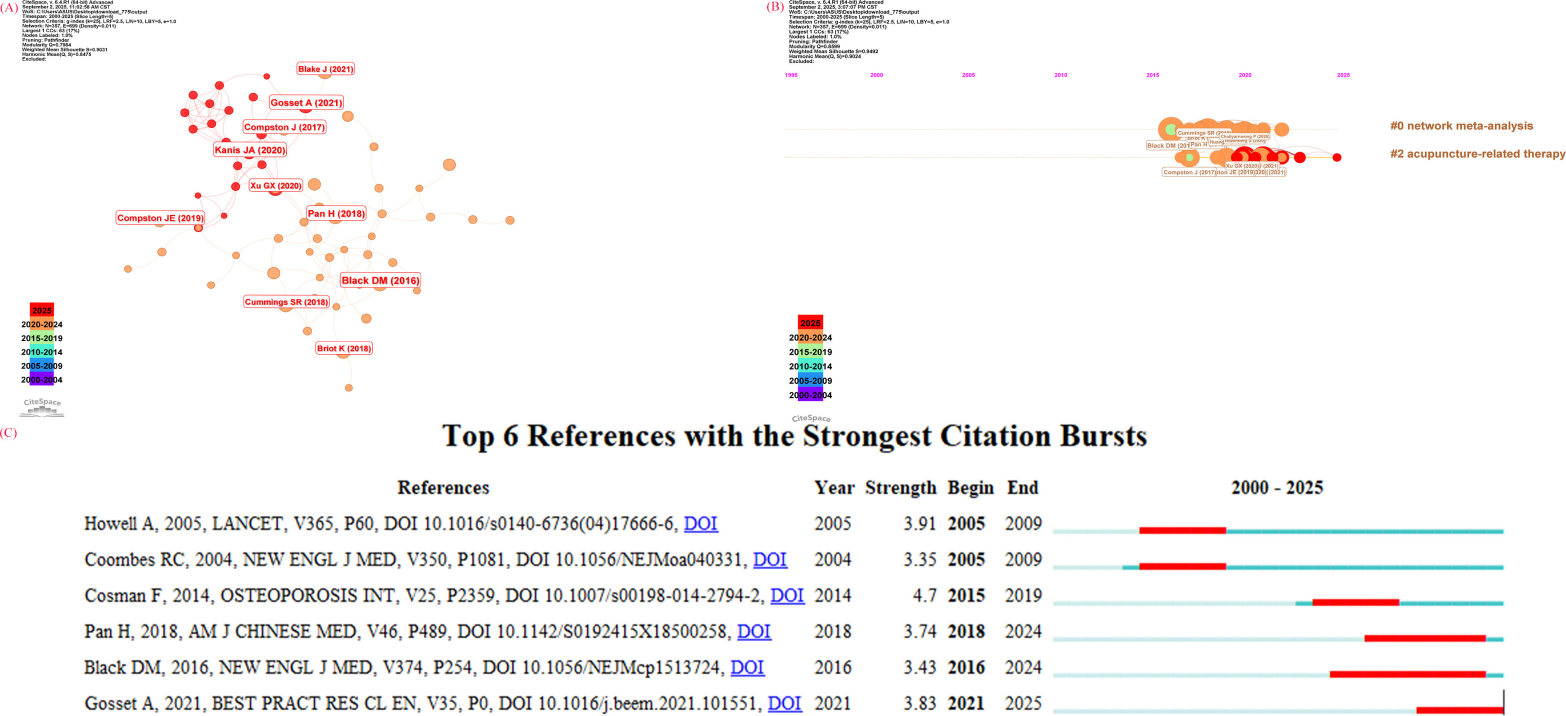
**(A)** Visualization graph of references of acupuncture for primary osteoporosis (CiteSpace). **(B)** A timeline view of acupuncture for primary osteoporosis. In the reference timeline profile, the later the timeline of each cluster appears, the more likely the cluster is to become a new research hotspot. **(C)** Reference with the strongest citation bursts of Acupuncture in primary osteoporosis.

Literature on the same topic is organized together, allowing researchers from various fields to easily locate relevant studies. For this study, CiteSpace 6.4.R1 was utilized to display the most highly cited literature in the field of acupuncture for primary osteoporosis (Figure 8A). The five articles with the highest number of citations are listed in Table 5. Among these, the most cited work, which has had the greatest impact on the field, was published in “Osteoporosis International” in 2014, with a total of 10 citations.

**TABLE 5:** Top five co-cited references in acupuncture treaty on primary osteoporosis.

Citation	First author	Year	Journal
2778	Cosman F (99)	2014	Osteoporosis International
955	Black DM (100)	2016	New England Journal of Medicine
335	Kanis JA (101)	2020	Osteoporosis International
130	Gosset A (54)	2021	Best Practice and Research Clinical Endocrinology and Metabolism
43	Pan H (41)	2018	American Journal of Chinese Medicine

The reference timeline profile (Figure 8B) was created by clustering the literature in chronological order. From Figure 8B, the intensity of acupuncture-related therapy indicates emerging hotspots in the field. This suggests that over time, researchers have increasingly emphasized the role of acupuncture in treating osteoporosis.

“References with citation busts” refer to articles that have been frequently cited over a specific period within a field. Figure 8C illustrates the citation bursts of the top six references concerning acupuncture for primary osteoporosis from 2000 to 2025. Among these articles, the most recently published literature is by Gosset et al. (54). Their team pointed out that hormone therapy can effectively improve bone loss within the first 2–3 years of menopause onset. Their findings have heightened researchers’ focus on elevating hormone levels during early menopause to mitigate osteoporosis.

## 4 Discussion

### 4.1 General information

In this study, we utilized the Web of Science Core Collection and employed CiteSpace 6.4.R1, VOSviewer 1.6.20, BICOMB, gCLUTO, Microsoft Charticulator, and SPSS 27.0 to comprehensively analyze the research status, hotspots, and trends related to acupuncture for primary osteoporosis from a macro perspective. A total of 775 relevant articles were ultimately retrieved. Over the past two decades, the number of publications in this field has increased annually, particularly since 2016. This trend indicates that research in this area has entered a phase of rapid development, characterized by emerging research hotspots. The most influential journal is *Osteoporosis International*, which focuses primarily on osteoporosis research. Currently, the United States holds the greatest influence in this field, largely due to osteoporosis-related research conducted in the U.S. or in collaboration with American institutions. The institution with the highest number of publications and greatest influence is Guangzhou University of Chinese Medicine. This institution is a medical school within the Chinese medical education system, dedicated to the study of Traditional Chinese Medicine and known for its unique acupuncture therapy, Jin’s Three-Needle Therapy (JTNT) (55).

Consequently, a significant volume of research has been produced in the field of acupuncture. The author with the highest number of publications is Bernard Cortet from the University of Lille, while the most cited author is J.A. Kanis from the University of Sheffield Medical School.

### 4.2 Research hotspots on acupuncture for primary osteoporosis

We conducted a comprehensive analysis and interpretation of the keywords and highly cited articles, categorizing the acupuncture for primary Osteoporosis into four primary domains: postmenopausal women, osteoporosis-related complications, Traditional Chinese Medicine, and the prevention of osteoporosis.

#### 4.2.1 Focusing on postmenopausal women

Menopause is a primary factor contributing to osteoporosis in women. After menopause, the decline in ovarian function causes estrogen levels to drop sharply. This leads to increased activity of osteoclasts (bone-resorbing cells), while the rate of new bone formation cannot keep pace with the rate of bone resorption. The result is a significant and rapid loss of bone mass, causing the microscopic structure of bones to become sparse and fragile, ultimately leading to osteoporosis. This condition is known as postmenopausal osteoporosis and is classified as a form of primary osteoporosis. Researchers are increasingly focusing on postmenopausal osteoporosis. In recent years, numerous articles have been published addressing fracture risk, prevention, quality of life, and treatment strategies for this condition. For instance, a systematic review published in 2022 (56) demonstrated that exercise effectively improves bone density and alleviates menopausal symptoms in postmenopausal women. In 2023, studies were published on fracture risk (57) and quality of life assessment (58) in postmenopausal osteoporosis. Research teams led by Stevenson (59) and Long et al. (60). Systematically reviewed preventive approaches for postmenopausal osteoporosis.

The focus of these studies was on regulating estrogen levels to improve postmenopausal osteoporosis. As early as 2016, an Asian study (61) demonstrated that acupuncture effectively alleviates conditions caused by estrogen deficiency, clearly highlighting its role in modulating estrogen levels.

Furthermore, the efficacy of acupuncture in treating postmenopausal-related conditions has been well demonstrated. Previous studies have confirmed that acupuncture improves symptoms such as postmenopausal overactive bladder (62) and hot flashes (63). This clearly indicates that, when treating postmenopausal osteoporosis with acupuncture, alleviating patient discomfort by addressing postmenopausal symptoms can enhance its therapeutic effect.

The findings of this study offer new insights for researchers investigating acupuncture treatment for primary osteoporosis. By examining the mechanisms through which acupuncture modulates estrogen levels, we can further explore additional therapeutic possibilities, particularly for postmenopausal osteoporosis.

#### 4.2.2 A study on the efficacy of traditional Chinese medicine in treating primary osteoporosis

Traditional Chinese Medicine therapies primarily include herbal medicine and acupuncture-related treatments. Recent studies have demonstrated that both approaches can produce significant therapeutic effects on primary osteoporosis.

First, traditional Chinese herbal medicine can provide significant therapeutic benefits for primary osteoporosis. Research conducted in 2023 indicated that acupuncture may influence the progression of osteoporosis by regulating the gut microbiome (16).

In addition, numerous clinical trials have been conducted in the field of acupuncture for primary osteoporosis, and the clinical effects have been substantiated. For instance, research by Deng et al. (64) has demonstrated that acupuncture can enhance bone mineral density in patients while also alleviating pain and other clinical symptoms to some extent. Additionally, Ping et al. (65) have shown that combining acupuncture with Traditional Chinese Medicine can effectively mitigate bone loss in early-stage menopausal women. Furthermore, clinical trials led by Ma et al. (66) have confirmed that acupuncture therapy plays a significant role in relieving patients’ pain and improving their overall quality of life.

Although recent animal studies have confirmed the efficacy of acupuncture in treating primary osteoporosis—such as the findings by Miao et al. (67) and colleagues, which demonstrated that acupuncture at specific acupoints can effectively promote bone formation in rats, and the research by Li et al. (68), which showed that acupuncture not only alleviates osteoporosis symptoms in ovariectomized rats but also improves their endocrine status—these results highlight the potential therapeutic benefits of acupuncture for osteoporosis.

We can conclude that traditional Chinese medical therapies, including herbal medicine and acupuncture-related treatments, effectively improve osteoporosis-related bone loss and slow disease progression. This effect is particularly evident when acupuncture is combined with herbal medicine, as supported by clinical trials and some animal studies. However, there is a lack of large-scale animal studies providing robust evidence to corroborate the results of clinical trials in this area. This gap highlights the need for researchers to design relevant animal experiments to generate stronger evidence-based findings.

#### 4.2.3 Focus on osteoporosis sequelae

Osteoporosis often results in numerous serious complications, the most severe of which are osteoporotic fractures.

Osteoporotic fractures, also known as fragility fractures, occur when decreased bone strength and increased brittleness due to osteoporosis cause fractures from minor trauma or even routine activities. These fractures are particularly prevalent among women aged 50–60 (69) and commonly affect areas with inherently lower bone mass or high weight-bearing loads, such as the lumbar spine (70), hip, pelvis, and wrist. Clinically, the Fracture Risk Assessment Tool (FRAX) (71) is widely used for evaluation. Lumbar spine fractures are the most frequent type of osteoporotic fracture (72, 73), typically presenting clinically as spinal pain.

Spinal pain, including spinal fractures, is a common clinical symptom of osteoporosis. For example, lumbosacral pain caused by osteoporosis frequently occurs in clinical practice, particularly among the elderly population (8). Additionally, spinal deformities resulting from osteoporosis can lead to abnormalities in cardiopulmonary and abdominal organ function, causing symptoms such as constipation, abdominal pain, abdominal distension, and loss of appetite. Acupuncture, known for its analgesic effects, is often considered an effective treatment for spinal pain associated with osteoporosis after symptom onset.

The results of this study indicate that acupuncture, as a non-pharmacological analgesic method in clinical practice (74, 75), has been used to treat primary osteoporosis by alleviating osteoporosis-induced spinal pain, particularly in the lumbosacral region. This area has been a significant focus of past research. Based on the findings presented in Figures 6C,D, it is evident that spinal rehabilitation is not only a current key research focus but also holds considerable potential for future investigation.

Although acupuncture has not demonstrated significant therapeutic effects on osteoporosis-related complications, including osteoporotic fractures, to date, it can alleviate patient suffering to some extent. Moreover, numerous clinical trials have shown that acupuncture effectively improves long-term symptoms and sequelae of various associated conditions. Examples include post-stroke depression (76), pregnancy-related vomiting (77), Parkinson’s disease-related insomnia (78), chronic sciatica due to lumbar disc herniation (79), chronic pain (80), and post-stroke dysfunction (81). These findings demonstrate that acupuncture’s long-term efficacy for various conditions and its ability to improve related sequelae are supported by substantial clinical evidence. Additionally, previous studies indicate that acupuncture can effectively improve osteoporosis-related sequelae, such as intestinal dysfunction caused by osteoporosis (82), and significantly enhance the long-term quality of life for osteoporosis patients (83).

The findings of this research indicate that investigators in the field of acupuncture treatment for primary osteoporosis are increasingly focusing on the sequelae caused by osteoporosis, achieving notable progress and producing numerous research outcomes. However, it is important to recognize that all current evidence regarding acupuncture’s efficacy for osteoporosis-related sequelae is derived from clinical trials. There remains a lack of animal studies to elucidate the specific mechanisms involved. Furthermore, research on acupuncture treatment for osteoporotic fractures—the most severe complication of osteoporosis—is currently non-existent. This highlights the need for researchers in this field to dedicate greater effort to investigating osteoporosis sequelae and the long-term efficacy of acupuncture.

#### 4.2.4 Research on osteoporosis prevention

With the passage of time and advances in technology, researchers’ focus on osteoporosis has gradually shifted from emphasizing treatment protocols to prioritizing disease prevention. This approach aims to prevent patients from developing osteoporosis, thereby effectively reducing its incidence. The foundation of bone health and strength lies in the continuous process of bone metabolism. When bone resorption exceeds bone formation, bone strength declines, leading to fractures. The loss of ovarian estrogen after menopause causes a significant decrease in bone strength, placing women at higher risk for osteoporosis. By identifying risk factors in postmenopausal women, the likelihood of future osteoporotic fractures can be assessed, enabling the implementation of appropriate preventive measures. All preventive strategies begin with bone-friendly lifestyle choices (6, 84). Preventing bone mass loss can be effectively achieved through daily calcium supplementation and other medications (85, 86), along with dietary adjustments (87). From a modern medical perspective, preventing osteoporosis through various approaches better maintains bone health, significantly reduces fracture risk, and fundamentally enhances the quality of life and independence of middle-aged and elderly individuals, particularly postmenopausal women (88–92).

Traditional Chinese Medicine (TCM) has always emphasized disease prevention, embodying the core principle of “treating disease before it manifests”—intervening before illness develops or worsens. Currently, TCM has demonstrated preventive effects against arthritis (93, 94), cardiovascular and cerebrovascular diseases (95, 96), and cancer (97, 98). However, definitive evidence supporting the preventive effects of TCM, particularly acupuncture, against osteoporosis remains limited. Our findings indicate that research on acupuncture for primary osteoporosis is increasingly focusing on its preventive role, suggesting that this area may experience a renewed surge in studies in the near future.

## 5 Conclusion

This study aims to explore the application of acupuncture in the treatment of primary osteoporosis from 2000 to 2025. It provides an overview of the current research status and offers predictions for future trends. The current research indicates that acupuncture has a wide range of applications in managing primary osteoporosis and demonstrates significant potential for further development. Despite certain limitations, this work may assist researchers in advancing acupuncture treatments for primary osteoporosis. Additionally, we have identified key future research priorities in this field. First, osteoporosis caused by menopause in women is emerging as a prominent research focus in acupuncture treatment for primary osteoporosis. Studies investigating how acupuncture alleviates postmenopausal symptoms by regulating estrogen levels have garnered considerable attention. Second, the role of traditional Chinese herbal medicine in treating osteoporosis should be emphasized, especially when combined with acupuncture therapy. Third, further research is needed to evaluate acupuncture’s effects on osteoporosis-related complications and its long-term therapeutic efficacy. Finally, we believe that research on acupuncture for osteoporosis prevention holds substantial promise.

## 6 Limitations

This study offers a comprehensive visual analysis of acupuncture for primary osteoporosis. However, several limitations should be considered.

First, the data sources for this study are relatively limited, as they are exclusively drawn from the WOSCC database and do not include other databases such as PubMed, Embase, or CNKI.

Secondly, the literature included in this study is limited to English. This restriction was implemented to prevent errors in the visualization software during analysis. Consequently, literature in other languages has been excluded.

Thirdly, the limitations of bibliometric software: The bibliometric software used in this study may overlook certain literature during analysis due to inherent constraints, which could potentially affect the final results to some extent.

## Data Availability

The original contributions presented in the study are included in the article/supplementary material, further inquiries can be directed to the corresponding authors.
